# Antimicrobial activity of carbon monoxide-releasing molecule [Mn(CO)_3_(tpa-κ^3^*N*)]Br versus multidrug-resistant isolates of Avian Pathogenic *Escherichia coli* and its synergy with colistin

**DOI:** 10.1371/journal.pone.0186359

**Published:** 2017-10-17

**Authors:** Jonathan Betts, Christopher Nagel, Ulrich Schatzschneider, Robert Poole, Robert M. La Ragione

**Affiliations:** 1 Department of Pathology and Infectious Disease, School of Veterinary Medicine, University of Surrey, Guildford, United Kingdom; 2 Institut für Anorganische Chemie, Julius-Maximilians-Universität Würzburg, Würzburg, Germany; 3 Department of Molecular Biology and Biotechnology, University of Sheffield, Sheffield, United Kingdom; Emory University School of Medicine, UNITED STATES

## Abstract

Antimicrobial resistance is a growing global concern in human and veterinary medicine, with an ever-increasing void in the arsenal of clinicians. Novel classes of compounds including carbon monoxoide-releasing molecules (CORMs), for example the light-activated metal complex [Mn(CO)_3_(tpa-**κ**^3^*N*)]Br, could be used as alternatives/to supplement traditional antibacterials. Avian pathogenic *Escherichia coli* (APEC) represent a large reservoir of antibiotic resistance and can cause serious clinical disease in poultry, with potential as zoonotic pathogens, due to shared serotypes and virulence factors with human pathogenic *E*. *coli*. The *in vitro* activity of [Mn(CO)_3_(tpa-**κ**^3^*N*)]Br against multidrug-resistant APECs was assessed *via* broth microtitre dilution assays and synergy testing with colistin performed using checkerboard and time-kill assays. *In vivo* antibacterial activity of [Mn(CO)_3_(tpa-κ^3^*N*)]Br alone and in combination with colistin was determined using the *Galleria mellonella* wax moth larvae model. Animals were monitored for life/death, melanisation and bacterial numbers enumerated from larval haemolymph. *In vitro* testing produced relatively high [Mn(CO)_3_(tpa-κ^3^*N*)]Br minimum inhibitory concentrations (MICs) of 1024 mg/L. However, its activity was significantly increased with the addition of colistin, bringing MICs down to ≤32 mg/L. This synergy was confirmed in time-kill assays. *In vivo* assays showed that the combination of [Mn(CO)_3_(tpa-κ^3^*N*)]Br with colistin produced superior bacterial killing and significantly increased larval survival. In both *in vitro* and *in vivo* assays light activation was not required for antibacterial activity. This data supports further evaluation of [Mn(CO)_3_(tpa-κ^3^*N*)]Br as a potential agent for treatment of systemic infections in humans and animals, when used with permeabilising agents such as colistin.

## Introduction

Antimicrobial resistance is a growing global concern, with clinicians in human and veterinary medicine faced with reduced therapeutic options to treat patients with infections caused by multidrug-resistant bacteria [1]. Gram-negative bacteria such as *Escherichia coli* have in recent years been highlighted as potential super bugs, due to increasing antibiotic resistance to many classes of antibiotic. This issue, added to the lack of antibacterials in the pipeline that specifically target Gram-negative bacteria, has left an ever-increasing void in the arsenal of clinicians, as the number of effective antibiotics declines [2]. Antibacterials of last resort such as carbapenems and polymyxins are increasingly used as front line drugs. However, resistance to carbapenems *via* the production of carbapenemases, efflux or reduced permeability has been widely reported [3]. This has led to the revival of previously abandoned antibiotics including colistin (polymyxin E), fosfomycin and chloramphenicol. With the increased use of these antibiotics, a significant rise in resistance has followed, recently noted with the discovery of MCR-1, the plasmidic colistin-resistance gene, in China [4–5] and in other countries in both humans and animals.

One potentially problematic group of pathogens are avian pathogenic *E*. *coli* (APEC). APECs make up part of the normal avian intestinal flora, but can cause serious clinical disease in poultry. Avian colibacillosis caused by APEC is an economically important infectious disease of domestic poultry. The aetiological agent responsible for colibacillosis is *Escherichia coli*, with the most commonly implicated serotypes being O1:K1, O2:K1 and O78:K80. Avian colibacillosis is a respiratory and systemic disease that exerts substantial welfare and economic costs on the poultry industry worldwide [6– 7]. Losses are incurred through mortality, condemnation of carcasses at slaughter, reduced productivity and costs associated with antibiotic treatment. Recent epidemiological evidence suggests that approximately 40% of mortalities from broiler flocks are associated with colibacillosis [8]. Avian colibacillosis is also responsible for up to 70% of mortality seen in broiler chicks 2–3 days after placement. Avian colibacillosis is a multifactorial disease and a number of risk factors are known, including prior or concurrent infection with respiratory viruses or *Mycoplasma*, stress and injury associated with formation of a social hierarchy, onset of sexual maturity and intense laying, as well as poor biosecurity, hygiene and ventilation [9]. Vaccination has proved successful for some APEC pathotypes, but the poultry industry is still reliant on antibiotics to treat APEC.

There are several reports suggesting that APECs harbour an array of resistance genes such as *bla*CTX-M-1, *bla*CMY-2 and *bla*TEM [10]. As APECs share identical serotypes and many virulence factors with human pathogenic *E*. *coli*, their potential as zoonotic pathogens should also not be underestimated [11, 12].

Alternatives to traditional antibacterials are investigated to supplement the growing need for antimicrobials. One group of potential drug candidates are carbon monoxide-releasing molecules (CORMs) such as CORM-3, which have received attention due to their effectiveness as antibacterial agents [13]. Several novel CORMs, including those activated by light at a specific wavelength such as [Mn(CO)_3_(tpa-κ^3^*N*)]Br have potential for therapeutic use against Gram-negative bacteria including *E*. *coli* [14]. Although the mechanisms of this compound are not entirely understood, the proposed mechanisms include membrane disruption due to hydroxyradical production, interference of metal ion uptake and inhibition of respiration, due to CO binding to respiratory cytochromes.

Recent work has also demonstrated the antibacterial action of a novel tryptophan manganese(I) carbonyl complex (Trypto-CORM) against *Neisseria gonorrohoeae*, which in recent years has also shown increasing antibiotic resistance [15]. Previous data suggests that the manganese-coligand core of the title compound does not reach the intracellular environment of bacteria [14]. However, using a polymyxin such as colistin, to permeabilise the outer membrane, could facilitate entry of the metal complex and increase the compound's antibacterial activity. The aim of the studies described here was to evaluate the *in vitro* and *in vivo* activity of the manganese complex [Mn(CO)_3_(tpa-κ^3^*N*)]Br alone and in combination with colistin against multidrug-resistance strains of avian pathogenic *E*. *coli*.

## Materials and methods

### Bacterial isolates, antibiotics and media

Avian pathogenic *E*. *coli* strains (*n* = 124) isolated from poultry farms across the UK were provided by Ridgeway Biologicals (Compton, UK). All strains were cultured on MacConkey agar for 16 h at 37°C, aerobically and characterised by biochemical and molecular profiles. Stock cultures were stored in glycerol at -80°C. [Mn(CO)_3_(tpa-κ^3^*N*)]Br (USC-CN028) was synthesised at the University of Würzburg, in the laboratories of Prof Ulrich Schatzschneider according to a published procedure [16]. Colistin sulfate powder was purchased from Cambridge Biosciences (Cambridge, UK). Mueller Hinton 2 agar and Mueller Hinton 2 cation adjusted broth was purchased from SigmaAldrich (Dorset, UK).

### Antibacterial assays

#### Antibiotic susceptibility testing

Antibiotic susceptibility testing of 24 commonly used antibiotics (ampicillin, ampicillin-sulbactam, azithromycin, aztreonam, cefepime, cefotaxime, cefoxitin, cephalexin, chloramphenicol, ciprofloxacin, clavulanic acid-amoxicillin, colistin, doxycycline, ertapenem, fosfomycin, gentamicin, imipenem, meropenem, nalidixic acid, nitrofurantonin, pipacillin-tazobactam, tetracycline, trimethoprim-sulfamethoxazole and tigecycline) was performed using the disc diffusion assay on Mueller Hinton 2 agar, against all 124 APEC strains, using standard methods previously described [17]. Plates were incubated at 37°C for 16 h, aerobically, after which zones of inhibition were recorded. Susceptibility/resistance was checked using breakpoints from the European Committee on Antimicrobial Susceptibility Testing [18]. A panel of strains resistant to ≥ 5 antibiotic classes were selected for further study in order to determine the minimum inhibitory concentrations (MICs) of [Mn(CO)_3_(tpa-κ^3^*N*)]Br and colistin.

#### Bacterial growth curves

Prior to MIC testing, growth curves were performed on the test bacterial strains with and without exposure to UV light (365 nm) for 2.5 min, equivalent to that required for activation of the Mn complex. This was to undertaken to confirm that any antimicrobial activity could be attributed to the Mn complex and not exposure to the UV light. To perform the growth curves, a 1/1000 dilution of a 16 h broth culture, equating to approximately 10^6^ CFU/mL was used for the starting inoculum. At set time intervals of 0, 2, 4, 6 and 24 h post inoculation, 100 μL samples were sampled, serially diluted and plated onto Mueller Hinton 2 agar. Colonies from the dilutions were enumerated after incubation at 37°C for 16 h, aerobically. Growth curves were plotted used GraphPad Prism 6.0 to check for differences in growth kinetics +/- UV exposure.

#### Minimum inhibitory concentrations

Minimum inhibitory concentrations of [Mn(CO)_3_(tpa-κ^3^*N*)]Br and colistin were determined alone and in combination against seven multidrug-resistant isolates and performed in 96-well microtitre plates using Mueller Hinton 2 broth. Assays were set up in checkerboard style with 2-fold decreasing concentrations of the Mn complex (512–0 mg/L) and colistin (8–0 mg/L) with a bacterial inoculum of 10^5^ colony forming units (CFU) per mL. Plates were incubated at 37°C, aerobically and checked for turbidity after 24 h. Where the MIC was not achieved, the dilution above the maximum dose was used for calculating the fractional inhibitory concentration indexes (FICIs). Fractional inhibitory concentration indexes were calculated using the following equation as previously described [19]. FICI = FIC of A (MIC of antibiotic A in combination with antibiotic B/MIC of antibiotic A alone) + FIC of B (MIC of antibiotic B in combination with antibiotic A/MIC of antibiotic B alone). FICI values of ≤ 0.5 suggest a synergistic interaction, > 0.5−1.0 as an additive effect, > 1.0 to < 4 as indifference and a value of ≥ 4.0 was classed as an antagonistic effect [20]. All experiments were performed in triplicate (biological repeats), and results are presented as mean values.

#### Kill-kinetic assays

Kill-kinetic assays were performed using strain 236/12. In brief, a 1/1000 dilution of a 16 h, aerobic culture, equating to approximately 10^6^ CFU/mL was used as the starting inoculum for each strain. To individual cultures, antimicrobials were added at final concentrations, which were as follows: [Mn(CO)_3_(tpa-κ^3^*N*)]Br (x 1 MIC), colistin (x 0.5/x 1 MIC) and the Mn complex-colistin combination (x 1 MIC–x 0.5 MIC). Cultures were incubated at 37°C under continuous agitation (225 rpm) for 24 h. At set time intervals of 0, 2, 4, 6 and 24 h post inoculation, 100 μL samples were collected, serially diluted and plated onto Mueller Hinton 2 agar. Colonies from the dilutions were counted after incubation, aerobically at 37°C for 20 h. Time−kill curves (CFU/mL vs time) were plotted using GraphPad Prism 6.0 software. Synergy was defined as bactericidal activity (≥2 log_10_ difference in CFU/mL) of the combination compared to the single agent after 24 h incubation. Unpaired student t-tests were performed to check for significant variance.

#### *In vivo* toxicity in *Galleria mellonella*

*In vivo* testing was conducted using the standardised *Galleria mellonella* invertebrate model (TruLarv^TM^, Biosystems Technology, Exeter). In brief, ten larvae were injected via the left proleg with 40, 200, 400, 600 mg/kg of Mn complex freshly prepared in sterile phosphate buffered saline (PBS) or a PBS control. Immediately after administration of the Mn complex, all larvae were placed in a sterile petri dish, illuminated with UV light (365 nm) (Uvitec LF206LS, Dutscher Scientific, Essex) for 2.5 min (Distance ~ 3 cm). The larvae then were incubated at 37°C for 48 h, with survival recorded (live/dead via a lack of response to touch) at 0, 24 and 48 h post treatment. The experiment was repeated with no exposure to UV light. All *in vivo* experiments were carried out in triplicate on 3 separate occasions.

#### Inoculum testing

To determine the optimum inoculum for larval killing (approx. 50% mortality of larvae at 24 h post infection), an inoculum test was performed. In brief, a 16 h culture of APEC strain 236/12 in Luria base broth was washed in PBS before being serially diluted in PBS. Colony forming units were determined by plating the dilutions on nutrient agar and incubating at 37°C for 24 h. Ten *G*. *mellonella* larvae were infected with the 16 h culture dilutions, equating to 10^3^, 10^4^, 10^5^ and 10^6^ CFU/larvae, via a 10 μL injection into the left proleg. Larvae were incubated at 37°C and scored for survival (live/dead) at 0, 24, 48, 72 and 96 h.

#### *G*. *mellonella t*reatment assays

Sixteen larvae were infected with 10^5^ CFU/larvae of APEC strain 236/12 via a 10 μL injection in a left proleg. Within 30 min after infection, a second injection into a right proleg was administered of the Mn complex (20 mg/kg in PBS), colistin (0.625 mg/kg), a combination of Mn complex and colistin (20 + 0.625 mg/kg) or PBS +/- UV exposure for 2.5 min at 365 nm post injection. Larvae were incubated at 37°C and scored for survival (live/dead) at 0, 24, 48, 72 and 96 h.

Melanisation scores for larvae were recorded over 72 h as an indicator of morbidity, based on a reversed scoring method previously published [21], whereby a score of 4 indicated total melanisation of the larvae, 2 equalled melanin spots over the larvae, 1 equalled discolouration of the tail and a score of 0 equalled no melanisation.

Bacterial enumeration from infected and treated larvae was performed over 24 h, by plating out larval haemolymph collected from larvae *post-mortem*. Larvae were injected with isolate 236/12 as described in the treatment assay and at 0, 2, 4, 6, 12 and 24 h, haemolymph from 3 larvae was collected. In brief, larvae were placed in cold storage (-20°C for 10 min) before being wiped with 70% ethanol. A sterile scalpel was used to remove the tip of the tail and haemolymph collected into an Eppendorf tube on ice. Haemolymph (100 μL) was serially diluted in sterile PBS and dilutions were plated out on MacConkey 3 agar (Oxoid, Basingstoke, UK). Plates were incubated for 18 h at 37°C, before bacterial enumeration was performed.

All assays were performed in triplicate and mean values presented. Survival curves, melanisation scores and bacterial counts were plotted using GraphPad Prism 6.0 software (San Diego, CA, USA). Analysis of survival curves was performed using the log rank test, with a *p* value of ≤ 0.05 indicating statistical significance [22]. Unpaired student t-tests were performed to check for significant variance in bacterial counts at 24 h.

## Results and discussion

Results from the antibiotic susceptibility testing indicated that 45 out of 124 APEC strains tested were multidrug-resistant (Table 1, S1 Table), with resistance observed against > 2 antibiotic classes in relation to breakpoints set by the European committee on antimicrobial susceptibility testing [18]. Of these strains 7 were resistant to > 5 antibiotic classes. No strains exhibited resistance to carbapenems or polymyxins.

**Table 1:** Phenotypic resistance of 124 avian pathogenic strains to commonly used antibiotics in relation breakpoints set by the European committee on antimicrobial susceptibility testing.

Antibiotic	Number of isolates showing phenotypic resistance	Percentage resistance
Ampicillin	49	39.5
Amoxicillim-clavulanic acid	45	36.3
Piperacillcin-Tazobactam	0	0
Ampicillin-Sulbactam	38	31
Cephalexin	6	4.8
Cefoxitin	3	2.4
Cefotaxime	4	3.2
Cefepime	1	0.8
Imipenem	0	0
Ertapenem	0	0
Meropenem	0	0
Aztreonam	4	3.2
Gentamicin	2	1.6
Amikacin	0	0
Tetracycline	85	68.5
Doxycycline	44	35.5
Tigecycline	1	0.8
Azithromycin	2	1.6
Nalidixic acid	31	25
Ciprofloxacin	4	3.2
Sulfamethoxazole/trimethoprim	23	18.5
Fosfomycin	1	0.8
Chloramphenicol	14	11.3
Nitrofurantonin	0	0
Colistin	0	0

**Fig 1:**
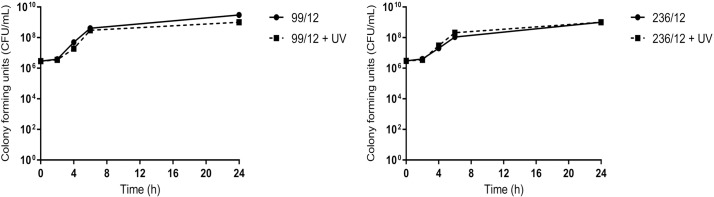
Growth curves of avian pathogenic *E*. *coli* strains 99/12 and 236/12 with and without exposure to UV light (365 nm) for 2.5 minutes.

Growth kinetics analysis from cultures with and out exposure to UV light (365 nm, 2.5 min) showed no significant difference (*P* >0.5) in growth rates or growth numbers at 24 h (Fig 1, S2 Table). This supported previous studies and confirmed that any antimicrobial activity would be due to the manganese carbonyl complex and not the UV light exposure [14].

Results from the broth microtitre testing revealed MICs of 1024 mg/L for [Mn(CO)_3_(tpa-κ^3^*N*)]Br on all isolates (Table 2, S3 Table). MICs of 1 mg/L to colistin were observed for all isolates. In combination, MICs of the Mn complex were significantly reduced (*P* < 0.05) in the presence of colistin, with MICs reduced to ≤ 32 mg/L. Calculation of FICIs indicate synergy was produced between [Mn(CO)_3_(tpa-κ^3^*N*)]^+^ and colistin (FICIs 0.129–0.281). It was found that UV activation (365 nm) had no impact on [Mn(CO)_3_(tpa-κ^3^*N*)]^+^ MICs in this study.

**Table 2:** Minimum inhibitory concentrations (MICs) of the Mn complex [Mn(CO)_3_(tpa-κ^2^*N*)]Br, colistin and combinations of both versus multidrug-resistant strains of avian pathogenic *Escherichia coli* and corresponding fractional inhibitory concentration indices (FICIs).

Strain no.	MIC (mg/L)		MIC in combination (mg/L)		FICI
	Mn complex	colistin	Mn complex + colistin	colistin + Mn complex	
102/12	1024	0.5	4	0.125	0.25
99/12	1024	1.5	0.5	0.25	0.17
100/12	1024	1	0.5	0.25	0.25
236/12	1024	1	0.5	0.25	0.25
176/12	1024	0.5	2	0.125	0.25
140/07	1024	1	32	0.25	0.281
16/12	1024	1	1	0.25	0.251

Relatively high MICs for the Mn complex are probably due to low membrane permeability of the entire compound. Previous research has shown that CO released from CORM readily reaches the intracellular environment, but the manganese-coligand moiety of the molecule does not, potentially due to lower permeability or lack of active transport into bacterial cells [14]. This could potentially limit the effectiveness of monotherapy with CORMs in medical and veterinary applications; thus, as demonstrated in the studies presented here combination therapy maybe a favourable option.

**Fig 2:**
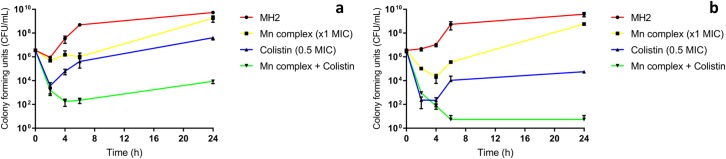
Time-kill curve of [Mn(CO)_3_(tpa-κ^3^*N*)]Br, colistin and combination of both agents (x1 MIC + 0.5 MIC) versus APEC strain a) 99/12 and b) 236/12 over 24 h.

Data from the kill-kinetic assays indicate that although antimicrobial activity was observed in microtitre assays, in a non-static model, regrowth in the Mn complex-treated bacteria was observed (Fig 2, S4 Table). The regrowth of cultures treated with the Mn complex 4 h post activation, suggests that the compound has a limited stability. This is potentially due to photodecomposition of the compound over time in ambient light. Colistin-treated cultures at 0.5 MIC produced good initial killing against both isolates tested, with regrowth observed after 2–4 h. Synergy observed in checkerboard assays of the CORM and colistin combination was confirmed in the kill-kinetic assays. The combination showed markedly (*P* < 0.05) increased killing activity when compared to either Mn complex or colistin alone, with ≥ 3 log_10_ CFU/mL difference at 24 h.

**Fig 3:**
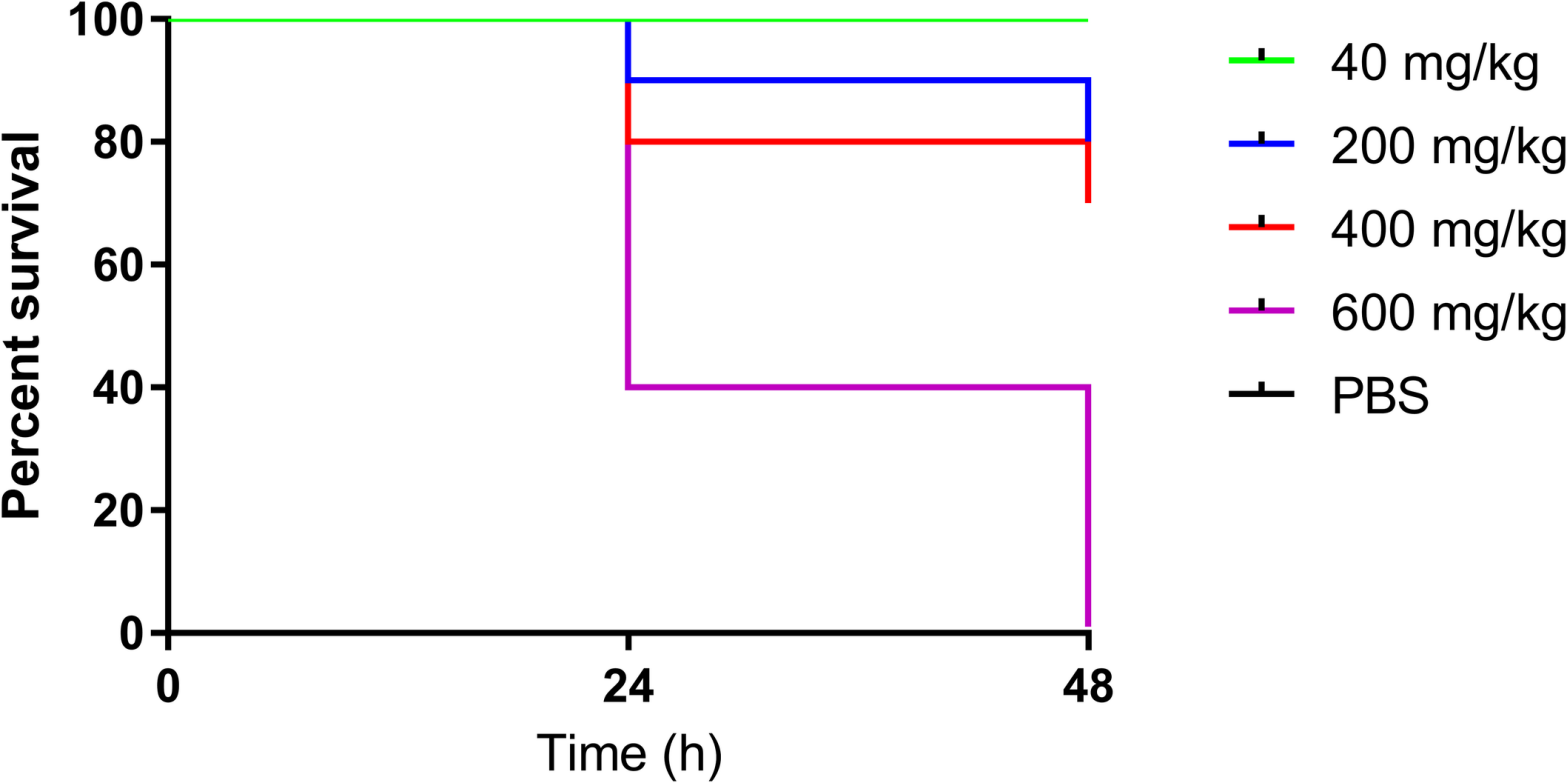
Survival curve showing *in vivo* toxicity of [Mn(CO)_3_(tpa-κ^3^*N*)]Br against *Galleria mellonella* over 48 h.

*In vivo* toxicity assays showed that *G*. *mellonella* survival numbers were reduced by 20% when exposed to 200 mg/kg of [Mn(CO)_3_(tpa-κ^3^*N*)]Br and by 30% when doses of 400 mg/kg were administered (Fig 3, S5 Table). Toxicity increased sharply when 600 mg/kg was administered, with 100% larval mortality. Importantly, doses of 40 mg/kg, double the concentration required in treatment assays, produced no toxicity from the Mn complex.

**Fig 4:**
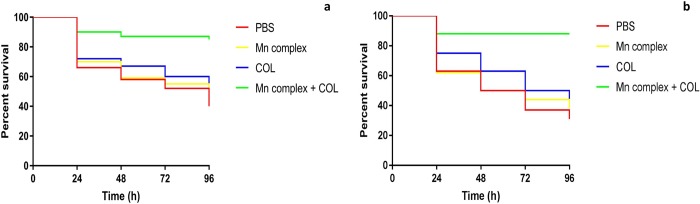
Survival curves (live/dead) of *G*. *mellonella* over 96 h after infection with 10^5^ CFU/larvae of APEC strain a) 99/12 and b) 236/12 and treatment with PBS, 20mg/kg of [Mn(CO)_3_(tpa-κ^3^*N*)]Br, 0.625 mg/kg of colistin, and combination of both agents.

*In vivo* treatment assays found that combination therapy significantly increased larval survival over 96 h compared to monotherapy (Fig 4, S6 Table). Little difference was observed between UV and non UV-exposed experiments, indicating that activation and perhaps CO alone, has little impact on the antibacterial activity in this model.

**Fig 5:**
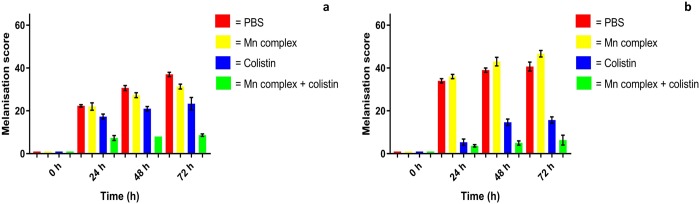
Melanisation assay in *Galleria mellonella* infected with 10^5^ CFU/larvae of APEC strain a) 99/12 and b) 236/12 and treated with PBS, 20 mg/kg of [Mn(CO)_3_(tpa-κ^3^*N*)]Br, colistin or a combination of both agents. Higher scores indicate greater insect morbidity.

High levels of morbidity were observed over 72 h with PBS- and Mn complex-treated larvae, with large melanisation scores recorded (Fig 5, S7 Table). Low melanisation scores were observed in larvae treated with colistin alone and the Mn complex-colistin combination, indicating these treatments reduce morbidity. Mn complex-colistin production significantly lower (*P* < 0.004) melanisation scores than treatment with PBS or monotherapy with the Mn complex or colistin. Alongside its antibacterial activity, colistin has been previously shown to possess potent anti-endotoxin activity [23]. This could explain the reduced ‘shock’ to the larval immune system/lower melanisation scores, in larvae treated with colistin or the Mn complex-colistin combination.

**Fig 6:**
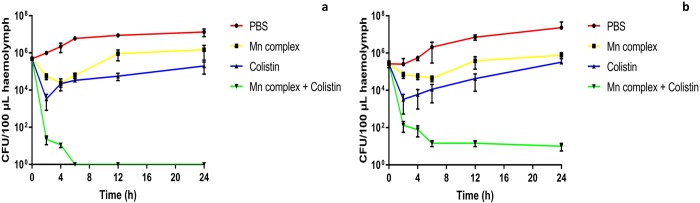
Bacterial counts over 24 h from *G*. *mellonella* post infection with 10^5^ CFU/larvae of APEC strain a) 99/12 and b) 236/12 and treatment with [Mn(CO)_3_(tpa-κ^3^*N*)]Br, colistin, a combination of both agents or PBS.

Bacterial counts over 24 h show that within larvae treated with PBS, bacterial numbers increased by >1.5 log_10_ CFU/100μL of hemolymph at 24 h (Fig 6, S8 Table). Treatment with colistin at 0.625 mg/kg initially reduced bacterial numbers of both 99/12 and 236/12, before regrowth of both isolates was observed at 2 h. However, bacterial numbers at 24 h did not reach that of 0 h. Treatment with [Mn(CO)_3_(tpa-κ^3^*N*)]Br produced initial bacterial killing, more significantly against 236/12, before regrowth was observed at 4 h, resulting in bacterial numbers at 24 h reaching the original CFU/larvae at 0 h. This would indicate that *in vivo*, the Mn complex produces bacteriostatic action within the first 24 h of administration. Bacterial counts indicate that the combination therapy was bactericidal and was significantly (*P* < 0.05) more effective in reducing bacterial numbers, than any monotherapy with [Mn(CO)_3_(tpa-κ^3^*N*)]Br, colistin or PBS, with total bacterial killing at 6 h for strain 236/12 and a reduction in CFU/100 μL of hemolymph to <1.5x10^1^ (Fig 6).

Although the mechanism of synergy remains to be determined, the observed activity is probably due to increased membrane permeability from the polymyxins, allowing greater amounts of the Mn complex inside the cell, thus increasing is ability to reach any potential target sites [24]. As the Mn complex also has the potential to distrupt bacterial membranes, overall membrane disruption would be greater in the combination, resulting in increased cell leakage and eventual cell death. Due to this duel attack on bacterial membranes, concentrations of colistin required for antibacterial activity are lower. Not only is this beneficial in terms of reduced toxicity to patients/animals, but also to combat resistance to polymyxins. Other membrane permeabilisers such as nonapeptide [25] could be investigated for efficacy as an alternative to colistin, from which there is a risk of nephrotoxicity.

Due to the original requirement of photoactivation to trigger the release of CO, it was assumed that the Mn complex would only serve as a model compound for fundamental studies on CO antibacterial activity, but with limited therapeutic applications. However, after investigation, it appears that its antibacterial action is not dependent on prior or post inoculation photoactivation *in vivo*. This fortuitous discovery, could increase the application of the compound from model system to the potential treatment of systemic infections in humans and animals.

In conclusion, we believe this is the first study to examine the *in vivo* antibacterial activity of a manganese carbonyl complex. Its activity against avian pathogenic *E*. *coli* (APEC) significantly increases when used in conjunction with colistin. The work presented here clearly demonstrates that the combination also significantly reduces APEC colony forming units within *G*. *mellonella* over 24 h and also reduces larval morbidity due to APEC infection. The *in vivo* data here also confirm the *in vitro* results presented and indicates that UV activation of [Mn(CO)_3_(tpa-κ^3^)*N*)]Br is not required for its antibacterial activity. Further work should examine the activity of the title compound against other important humans and animal pathogens, such as *Acinetobacter baumannii*, *Pseudomonas aeruginosa* and *Staphylococcus aureus*, which are also common carriers of antibiotic resistance genes and are associated with serious infections. It is clear that [Mn(CO)_3_(tpa-κ^3^)*N*)]Br and related compounds have potential for future applications in human and veterinary medicine. Furthermore, it appears that the antimicrobial activity of metal-carbonyl complexes does not necessarily always is based on the CO release efficiency [26].

## Supporting information

S1 Table Raw antibiogram data for 124 avian pathogenic *E. coli* strains. (XLSX)

S2 Table Raw data for UV (365 nm) exposure effect on avian pathogenic *E. coli* growth rate. (XLSX)

S3 Table Raw minimum inhibitory concentrations data against avian pathogenic *E. coli* strains. (XLSX)

S4 Table Raw kill kinetics data for [Mn(CO)_3_(tpa-κ^3^*N*)]Br, colistin and combination of both agents (x1 MIC + 0.5 MIC) versus APEC strains 99/12 and 236/12 over 24 h. (XLSX)

S5 Table Percentage survival scores for *G. mellonella* toxicity assay. (XLSX)

S6 Table Pooled percentage survival scores for *G. mellonella* live/dead treatment assays [Mn(CO)_3_(tpa-κ^3^*N*)]Br, colistin and combination of both agents. (XLSX)

S7 Table Melanisation scores for *G. mellonella* treatment assays with [Mn(CO)_3_(tpa-κ^3^*N*)]Br, colistin and combination of both agents. (XLSX)

S8 Table Bacterial counts (colony forming units/100μL of haemolymph) over 24 h after treatment [Mn(CO)_3_(tpa-κ^3^*N*)]Br, colistin and combination of both agents. (XLSX)
